# Distributing Agency and Experience in Therapeutic Interaction: Person References in Therapists' Responses to Complaints

**DOI:** 10.3389/fpsyg.2021.585321

**Published:** 2021-04-21

**Authors:** Marja Etelämäki, Liisa Voutilainen, Elina Weiste

**Affiliations:** ^1^Faculty of Humanities, University of Helsinki, Helsinki, Finland; ^2^Faculty of Humanities, University of Oslo, Oslo, Norway; ^3^Faculty of Social Sciences, University of Helsinki, Helsinki, Finland; ^4^Finnish Institute of Occupational Health, Helsinki, Finland

**Keywords:** agency, person reference, conversation analysis, interactional linguistics, psychotherapy interaction, Finnish

## Abstract

The primary means for psychotherapy interaction is language. Since talk-in-interaction is accomplished and rendered interpretable by the systematic use of linguistic resources, this study focuses on one of the central issues in psychotherapy, namely agency, and the ways in which linguistic resources, person references in particular, are used for constructing different types of agency in psychotherapy interaction. The study investigates therapists' responses to turns where the client complains about a third party. It focuses on the way therapists' responses distribute experience and agency between the therapist and the client by comparing responses formulated with the zero-person (a formulation that lacks a grammatical subject, that is, a reference to the agent) to responses formulated with a second person singular pronoun that refers to the client. The study thus approaches agency as situated, dynamic and interactional: an agent is a social unit whose elements (flexibility and accountability) are distributed in the therapist-client interaction. The data consist of 70 audio-recorded sessions of cognitive psychotherapy and psychoanalysis, and the method of analysis is conversation analysis and interactional linguistics. The main findings are that therapists use the zero-person for two types of responses: affiliating and empathetic responses that distribute the emotional experience between the client and the therapist, and responses that invite clients to interpret their own experiences, thereby distributing control and responsibility to the clients. In contrast, the second person references are used for re-constructing the client's past history. The conclusion is that therapists use the zero-person for both immediate emotional work and interpretative co-work on the client's experiences. The study suggests that therapists' use of the zero-person does not necessarily attribute “weak agency” to the client but instead might strengthen the clients' agency in the sense of control and responsibility in the long term.

## Introduction

One of the prime reasons clients request psychotherapy is their experience of a loss of agency in life (Wahlström, 2006). Clients may feel that their ability to attribute thoughts, feelings and actions to themselves, to control their own actions, and to influence their own choices is severely restricted (e.g., Avdi, 2005). The various psychotherapeutic traditions use different methods for seeking to help clients develop their diminished agency. For instance, in humanistic therapies, introspective reflections in a supportive environment are thought to empower clients to become more self-determining, while, in behavioral therapies, gaining new skills is seen as a means of increasing clients' agency by providing more options for acting (Williams and Levitt, 2007). By contrast, in therapies that draw on social constructivism, agency is understood to be negotiated and constructed in clinical interaction (Avdi, 2005). Therapist responsiveness is understood to facilitate the joint construction of new interpretations of previous experiences as well as new meanings attached to previously used words. This is thought to increase clients' ability to adopt a reflective position toward their experiences and that in way diminish non-agentic positioning (Avdi et al., 2015). This framework thus emphasizes the role of linguistic practices in the process of displaying and diminishing clients' non-agentic positioning of themselves (e.g., Avdi, 2005; Toivonen, 2019).

According to previous research, one linguistic practice for non-agentic positioning is the use of “agentless” talk, i.e., the “avoidance” of personal reference forms (Kurri and Wahlström, 2007). When clients use obscure personal forms, therapists often use specific person references (Kurri and Wahlström, 2007), in particular the second person singular pronoun, to invite them to move from a non-agentive to a more agentive and responsible position. Nonetheless, it is not only clients who use impersonal forms when referring to themselves; occasionally, therapists also use impersonal forms when referring to clients. Kurri and Wahlström (2007) studied one therapist's use of agentless formulations and found that the therapist treated the client's agentless formulations as a delicate matter and used agentless formulations as a step-by-step strategy when working toward agentic reformulations. They suggested that the therapist's use of agentless formulations is a strategy for saving the client's face (Kurri and Wahlström, 2007).

In our study, we explore therapists' use of impersonal forms, in particular the zero-person construction, when referring to clients. We compare them to turns in which therapists refer to the client with a second person singular pronoun. In our analyses, we draw on interactional linguistic studies on the use and meaning of Finnish personal forms in everyday informal interaction (Laitinen, 2006; Visapää, 2008) and interactional and anthropological studies on agency (Enfield and Kockelman, 2017). The aim is to gain understanding of the ways in which these two different personal forms (the zero-person and second person singular) are used in psychotherapy interaction, given that personal forms allow for the distribution of agency in various ways (see e.g., Couper-Kuhlen and Etelämäki, 2015).

Recent studies of language and social interaction suggest that agency is dynamic and social and show that agency can be distributed in different ways in interaction (see e.g., Enfield, 2013; Enfield and Kockelman, 2017). According to these studies, agency is rarely the possession of single individuals; rather, over a course of action, agency can be distributed in such a way that the individuals involved play more or less complementary roles in performing the action. Moreover, multiple individuals can be joined in a single unit of motivation and accountability. Agency in interaction is, thus, understood as “a fission-fusion affair involving constant navigation of separateness and boundedness, affiliation and disaffiliation, an endless tacking back and forth between inhabiting different social units, with always-relevant consequences for our social relationships, both fleeting and enduring” (Enfield, 2013, XVI). In this study, we adopt this view of agency. Thus, we understand agency in two different ways. First, we understand agency as flexible, social and distributed (Enfield, 2017): an agent is a social unit whose elements (flexibility and accountability) are dynamically distributed in real-time interaction between the therapist and the client. Second, agency can refer to clients' ability to take initiative and responsibility for their actions in everyday life, which is a more traditional view of agency in psychotherapy. In this study, we focus on the dynamic distribution of agency between the therapist and the client in the therapy session, and extend the idea of distributed agency to emotion and experience. We analyze how the client's earlier emotions and experiences are constructed in the psychotherapy interaction either as shared or non-shared by the client and the therapist, that is, either as epistemically accessible to both of the participants or not. We analyze the dynamic construction of agency and experience through the use of personal forms in Finnish psychotherapy interaction. We use this linguistic phenomenon as a concrete and observable example of negotiating and constructing agency *in situ*. By focusing on the therapist's responsive turns, we discuss the ways in which such turns both share and support the client's agency. In what follows, we begin by shortly introducing the Finnish zero-person construction and the previous research on the topic in the context of psychotherapy and everyday interaction. This will form the basis for our analysis, presented in the section Results.

## Zero-Person in Previous Research

Due to their different grammatical structures, in particular person systems, different languages possess different affordances for distributing agency and experience in interaction. In the following, we first provide a brief overview of the Finnish person system with a focus on the impersonal forms, in particular on the form called “zero-person.” We then give a brief review of the ways the zero person has been described in previous research on psychotherapy. Lastly, we demonstrate how the zero-person is used for distributing agency and experience in everyday informal Finnish interaction because this will provide the basis for our analysis of the use of the zero-person in psychotherapy interaction.

As in many other languages spoken in Europe, Finnish features personal pronouns for expressing the first, second and third person in both the singular (SG) and plural (PL): *minä* (“I”), *sinä* (“you.SG”), *hän* (“she/he”), *me* (“we”), *te* (“you.PL”), *he* (“they.PL”). Finnish is, however, distinct in that in addition to the first, second and third person singular and plural form, it features a personal passive and a zero-person. In Finnish, the personal passive form always implies a human agent. For example, the passive clause *poikaa lyötiin kivellä* (“the boy was hit by a stone”) implies that the boy was struck by a stone thrown by a human agent or agents. In addition to the personal passive, however, Finnish possesses a zero-person construction (marked as Ø in the translation lines of the transcripts). The zero-person construction has no overt subject, and the predicate verb appears in the third person singular form:


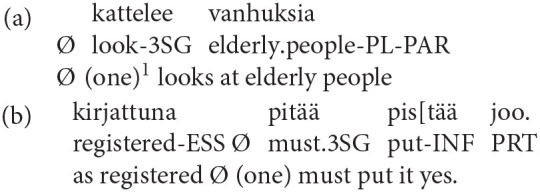


The difference between the Finnish personal passive and the zero-person is that whereas the passive refers to a collective agent, the zero-person refers to an individual but unspecified agent (Laitinen, 2006). Depending on the context, the zero-person can refer to either one of the speech act participants (first, second, or third person), in other words, it can be interpreted as “anyone,” “I,” or “you.”

In earlier research on Finnish, the zero-person was first analyzed as a “missing person.” Its use was claimed to be a negative politeness strategy to avoid explicit personal reference. Based on conversational data, newer research has, however, criticized this view and suggested that in affective contexts, the zero-person offers an indexical site to be identified with, “an empty place of the common experience, constructed for anyone to enter” that invites the recipient to view or experience the world from that place (Laitinen, 2006, 218; see also Laitinen, 1995). Moreover, in the context of directives, it has been suggested that it is employed as an offer to distribute agency between the participants more evenly than in requests with explicit personal forms (Couper-Kuhlen and Etelämäki, 2015). Research on Finnish psychotherapy interaction has largely adopted the former view.

Prior research on psychotherapy has suggested that the grammar of verbs plays a key role in mediating linguistic constructions of personal agency (Todd 214). It has been argued that clients who feel they are in an object position with respect to the difficulties they are facing use stative verbs (such as *have* a problem, *is* depressed) to display their problems (Todd, 2014). Previous research on Finnish psychotherapy has suggested that the agent of a particular action is typically left unspecified by using, for instance, zero-person verb forms (Kurri and Wahlström, 2007; see also Toivonen, 2019). According to these studies, clients use the zero-person form in at least two types of interactional contexts. First, clients employ it to diminish their personal responsibility (Kurri and Wahlström, 2007). By using the zero-person, clients can present themselves as victims, as people who lack control over the actions they are describing. The zero-person form is also typically used when clients describe themselves as objects or stooges of someone else's actions (Toivonen et al., 2019). In this case, the other actor can be anything that is referred to as initiating the action or creating the client's circumstances, such as a diagnosis, divorce or childhood events (Toivonen et al., 2019).

These types of expressions are noted to provide clients with a strategy to save face as a moral person when describing, for instance, their alcohol use, drunk driving or other presumably morally questionable behavior (Kurri and Wahlström, 2007; Halonen, 2008). Prior research has shown how therapists point out, challenge and reformulate such expressions (e.g., Kurri and Wahlström, 2007; Partanen et al., 2010), as it is their therapeutic task to place the client in an agentic position in his or her life (Kurri and Wahlström, 2007). Second, the zero-person form enables clients to discuss their own experiences in a way that constructs the experiences as commonly sharable (Halonen, 2008). By leaving the reference open, clients can invite others to identify with their description. Therapists, in turn, have been found to use the zero-person construction to show that the client's description is typical or general, for instance, for all addicts in group counseling (Halonen, 2008). The zero-person can also function as a face-saving strategy by not defining whose experience is in question: it enables discussion on difficult issues without pointing a finger at the client (Kurri and Wahlström, 2007; Halonen, 2008).

As mentioned at the beginning of this section, interactional linguistic research on the Finnish zero-person has largely rejected the view that the zero-person is used as a face-saving or negative politeness strategy because these theories are not directly compatible with an interactional view on language and interaction (see also Schegloff, 1988). Instead, it has suggested that zero person forms are typically found in two types of contexts: in affective accounts, and directives. In affective accounts, it invites the recipient to share the experience and the stance with the teller (e.g., Laitinen, 1995, 2006; Visapää, 2008) as in Example (1):


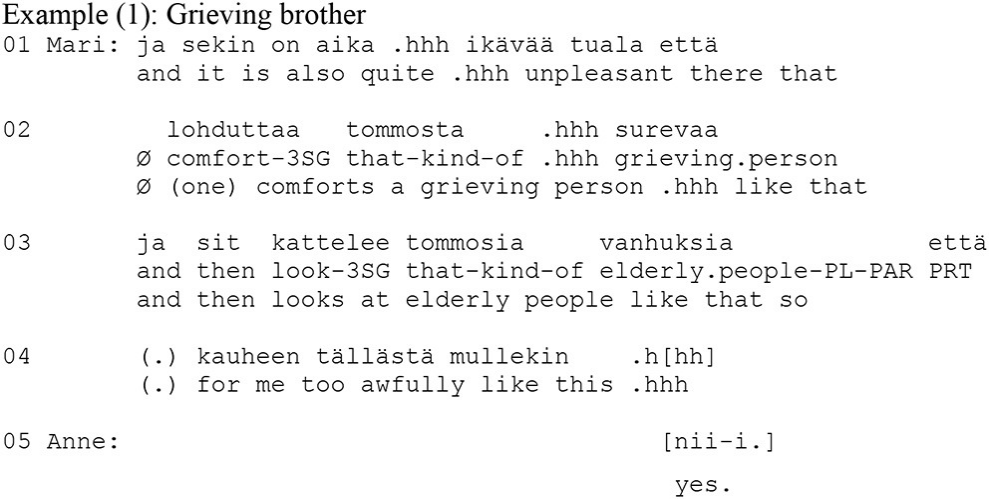


In affective contexts, it is thus suggested that the zero-person form is used for inviting the recipient to share an emotional stance toward the experience (Laitinen, 2006; Visapää, 2008). In other words, it can be used for distributing the experience. In the context of directives, on the other hand, the zero can be used for negotiating the agency of the future action with respect to responsibility and accountability for the action. It offers to distribute agency more evenly between the participants than the 1st and 2nd person forms (Couper-Kuhlen and Etelämäki, 2015; see also Rossi and Zinken, 2016 on similar phenomena in Italian and Polish). This is exemplified in the following Example (2). The example comes from a telephone call where the reason for the call is that Satu (who lives in Northern Finland) has forgotten her wallet in Vesa's (who lives in Southern Finland) car during Vesa's visit to Northern Finland. Satu now calls Vesa in order to ask him to send her the wallet by mail as a registered letter.


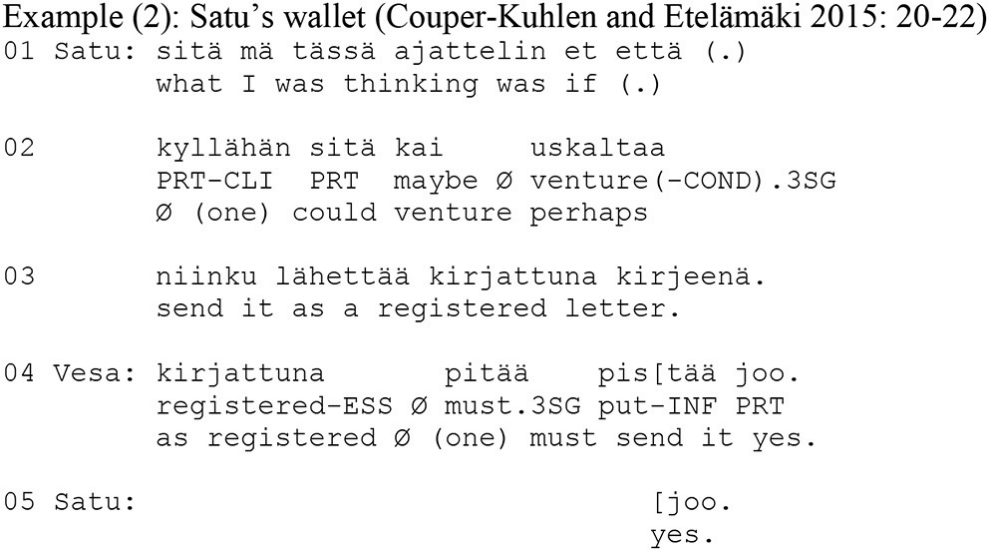


Satu's request (lines 1–3) is formulated as a declarative statement including a modal verb *uskaltaa* (“venture”) with a zero-person construction. Correspondingly, Vesa's response is formulated as a declarative statement that includes the modal verb *pitää* (“must”) with a zero-person construction. The modal verb *pitää* (“must”) in Vesa's turn expresses stronger necessity that the one in Satu's turn, and by that means Vesa displays independence in evaluating how the wallet will be best sent. Although it is clear that Vesa will perform the future action, both participants thus participate in deciding upon that action. In turns involving directions that are formulated with a zero-person construction, the zero-person occurs typically together with modal verbs that express the necessity/desirability of the proposed action (e.g., “can,” “need,” “must”). These turns are, moreover, declaratively formulated; i.e., they are formulated as statements that invite the recipient first to evaluate the rationale behind the action as well as the manner of the performance of that action. By offering the recipient of a directive a place to co-evaluate the necessity/desirability and manner of performance of an action, the combination of the zero-person and a modal verb distributes agency—accountability and responsibility over the action—between the participants more symmetrically than first or second person forms.

In the following, we explore psychotherapists' use of person reference forms. More specifically, we investigate how the use of the zero-person and second person singular pronoun distributes the client's experience and agency in the therapist's talk and how the choice of personal form corresponds to the action that the turn is accomplishing.

## Data and Methods

The data consist of 70 audio-recordings of actual psychotherapy encounters in Finland, collected in 1999–2009 in two different private sector clinics. The data come from four different dyads: two therapists with four different clients. One therapist is an experienced psychoanalyst, representing an object-relations-oriented psychoanalytic school. The other therapist is an experienced cognitive therapist, representing a cognitive-constructivist strand of cognitive therapy. The lengths of the encounters vary from 45 to 60 min and comprise ~30 h of interaction from both therapy approaches. The data are transcribed according to the transcription conventions developed by Jefferson (2004). Both clients in cognitive therapy were women in their twenties suffering from depression. One suffered also from panic attacks, while the other had been diagnosed with a personality disorder. In psychoanalysis, one of the clients was a man in his forties suffering from depression and work-related burn-out. The other client was a woman in her sixties experiencing a difficult situation in her life (her husband was terminally ill). In cognitive therapy, regular planned meetings were held approximately once a week. In psychoanalysis, the frequency of the sessions was approximately three times a week.

Informed consent was obtained from all the participants for the publication of any potentially identifiable images or data included in this article. The therapists informed the clients of the research, both verbally and in writing. They were also given the possibility to withdraw their consent at any point of the data collection. The researchers were not present in any of the therapy encounters. The anonymity of the therapists and clients has been carefully ensured: all names and other details which might enable identification of the participants have been altered in the text and data excerpts.

The data were analyzed by means of conversation analysis and interactional linguistics. Conversation analysis is a systematic method for studying human social interaction. According to conversation analysis, social actions are accomplished through adjacent utterances (Schegloff, 2007; Stivers and Sidnell, 2012; Clift, 2016). This means that a prior utterance constrains the following turn, which shows what social action the prior utterance was understood to be; for instance, questions elicit answers, formulations elicit confirmation or rejection, and the sharing of an emotional experience elicits affiliation. Interactional linguistics is the conversation analytically informed study of linguistic structure and meaning, the starting point of which lies in understanding language as a thoroughly interactional phenomenon (Couper-Kuhlen and Selting, 2018).

The centrality of sequences of adjacent actions and linguistic structures has some important implications for conversation analytic studies of psychotherapeutic interaction. Thus, phenomena that are specifically relevant for psychotherapy, such as the therapist-client relationship and the expression of emotions in interaction, are examined in the context of sequences of actions, for example clients' descriptions of their experience and therapists' formulation of that experience (Peräkylä et al., 2008; Peräkylä, 2012). Conversation analysis and interactional linguistics assume that interpersonal relations, emotions and the like exist in and through sequences of actions. Consequently, the aim of conversation analytic studies in psychotherapy is to describe not only these actions but also the way psychotherapeutic processes occur through sequences of such actions (Peräkylä, 2019). In this study, we focus on one aspect of that therapeutic process, the distribution of the client's experience and agency and describe how it is accomplished through the therapist's choice of the personal form. Rather than analyzing this linguistic form (person reference) as such, we use the results of previous interactional linguistic studies to analyze how agency is distributed in psychotherapy interaction.

Our analysis began by first collecting every sequence of actions in which the client complained about a third party, for instance, their mother or spouse. We decided to restrict our analysis in this context because previous studies have shown that complaining about a third party is a problematic activity in everyday interactions (see Heinemann and Traverso, 2009), and both the zero-person form and the second person form were found to be used in this context (see Voutilainen et al., 2010a). In these complaints, clients present themselves as having been inappropriately treated by the third party in question and describe the negative experiences they have encountered with that person (Voutilainen et al., 2010a). From 60 h of interaction, 74 such third-party complaints were identified. Next, we analyzed the therapists' responses to the clients' third-party complaints and investigated the ways in which these responses addressed the clients' accounts. At this point, we paid specific attention to the person reference forms that the therapists used in their responses. On the therapists' responses to the 74 third-party complaints in our data, 51 were formulated with a zero-person, and 23 with a second person singular pronoun. The cases were divided into three categories based on the immediate sequential context and the personal reference form (zero-person or second person singular) used in the therapist's turn. These categories were (1) a zero-person form when displaying empathy (32 cases), (2) a zero-person form when inviting an interpretation (19 cases), and (3) the second person form in the context of tracing the problematic elements of the client's life history (23 cases). In the Results section below, we present these categories through four data examples.

## Results

In the therapists' responses, we found that the zero-person form occurred in two contexts: in displays of empathy toward the client's emotions and in interpretations of the client's experiences and circumstances. In turn, the second-person singular pronoun was used when the therapist was re-constructing the client's history. In the following, we first discuss the use of the zero-person and then compare these uses to cases where the therapist uses the second person singular pronoun.

### Empathetic Use of the Zero-Person: Treating the Experience as Actual Here and Now

We found that the zero-person form treats the client's experience as actual and epistemically available in the here-and-now of the therapeutic situation or in the client's current life more generally. As has been demonstrated in everyday talk (Laitinen, 1995, 2006), the use of the zero-person presents an experience as shareable and thus epistemically available to the recipient. In therapeutic interaction, the therapist can project a sense of speaking “from within” the experience when using the zero-person (Vehviläinen, 2003; Voutilainen et al., 2010b; Weiste et al., 2016). In other words, the zero-person is used for displaying empathy: for displaying recognition and understanding of the client's emotional experience as expressed by the client.

Extract (3) below is drawn from a cognitive psychotherapy session. It represents an example of the use of the zero-person in a turn functioning as an empathetic response. The client discusses her persistent fear of being physically assaulted when out in the city (lines 5–11). This fear is particularly intense during the night, even when the client is with her boyfriend, Ville (lines 16–20, 23). The client's talk is hesitant and perturbed: it includes several pauses, and self-initiated same-turn repairs of different types (see Schegloff, 2011).


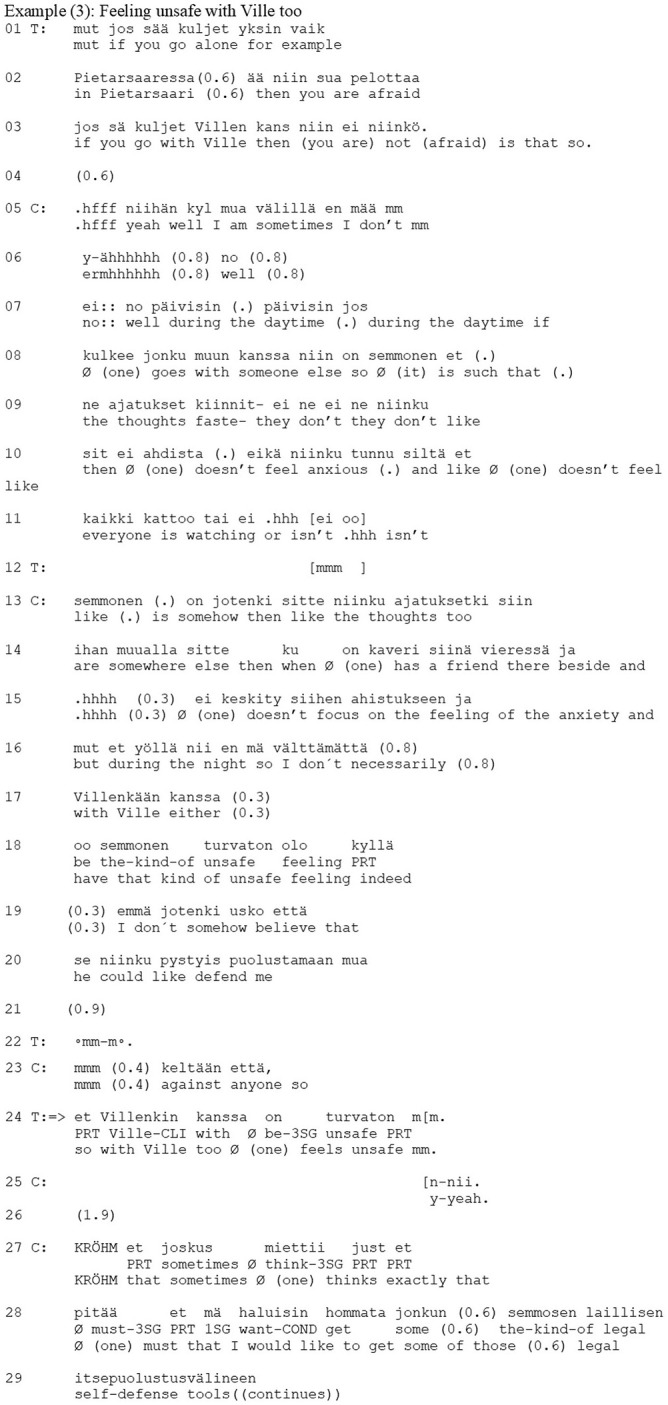


In her account in lines 5–24, the client uses the zero-person form (lines 7–8, 10, 13–15) to display her experience as shareable (Laitinen, 1995, 2006). In line 24, the therapist takes the turn and produces a formulation that highlights the key descriptive element of the client's account (from lines 16–20, 23) (Weiste and Peräkylä, 2013): the client is afraid, even when accompanied by her boyfriend. The therapist's turn begins with the particle *et* (“that”), which marks it as a paraphrase of the client's previous turn. The emotion (feeling unsafe, literally translated “is unsafe”) is picked up from the client's previous turn. Like the client in her account (lines 7–8, 10, 13–15), the therapist uses the zero-person form of the verb in the formulation (“Ø is”). It is noteworthy that whereas in lines 16–18 the client begins to formulate the utterance as a negative statement but self-interrupts and reformulates it as a positive one, the therapist's formulation is constructed as a positive statement. Moreover, whereas at the end of her account (lines 16–17) the client uses the first-person form *emmä välttämättä Villenkää kanssa* (“I don't necessarily [feel safe] with Ville either”), the therapist uses the zero-person in her formulation. The therapist's formulation with the zero-form does not, thus, merely echo the agency and experience in the client's account. Instead, the therapist reformulates the client's account based on the self-repair in the client's utterance, and the zero-person offers the client a place as an experiencer of this re-formulated account (see Laitinen, 2006).

On completion of her formulation (line 24), the therapist produces the particle *mm* which conveys acknowledgment and confirmation, and by producing it at this point, the therapist positions herself as the recipient of her own formulation. Thus, she orients to her formulation as if it were the client's words that she receives. By using the zero-person form, the therapist can be heard to speak “from within” the client's experience (Vehviläinen, 2003, Voutilainen et al., 2010b, Weiste et al., 2016), thereby distributing the agency of the experiencing subject between the therapist and the client. The use of the zero person creates a place for an experience that is shared, not through lived life but through empathetic imagination. The therapist therefore treats the experience as available and understandable as such, and, in that way, she avoids implying that the client needs to provide a further account of the experience. The client confirms the therapist's formulation (*nii*, “yeah,” line 25), and nevertheless elaborates on the feeling (lines 27–29) by using the zero-person herself, thus continuing to treat the experience as mutually shared.

In this example, both the client and the therapist use the zero-person to co-describe the client's experience, which has been made mutually available during the therapy session. The therapist's use of the zero-person displays empathetic stance to the client's account (see also Voutilainen et al., 2010b, Weiste et al., 2016). By treating the client's experience as epistemically available, the therapist makes shared experience possible. In other words, through empathetically formulating the client's words, the therapist, as it were, participates in the client's experience.

### Interpretive Use of the Zero-Person: Analytical Distancing From the Experience

Another context where therapists use the zero-person is when interpreting the client's experience. Example (4) shows a case in point from a psychoanalytical therapy session. Prior to the example, the client has been discussing the time in her childhood when she lived with her mother. She describes herself as an obedient girl who always attempted to comply with her mother's wishes. Moreover, she constantly felt that she was “necessary, yet not very important” to her mother. Prior to the extract, the client has hesitantly suggested that her mother was quite unreliable for a child, and the therapist has pointed out that it is difficult for the client to say anything bad about her mother. At the beginning of the extract (lines 1–15, 18–20), the client elaborates further on her experience of her mother's unreliability.


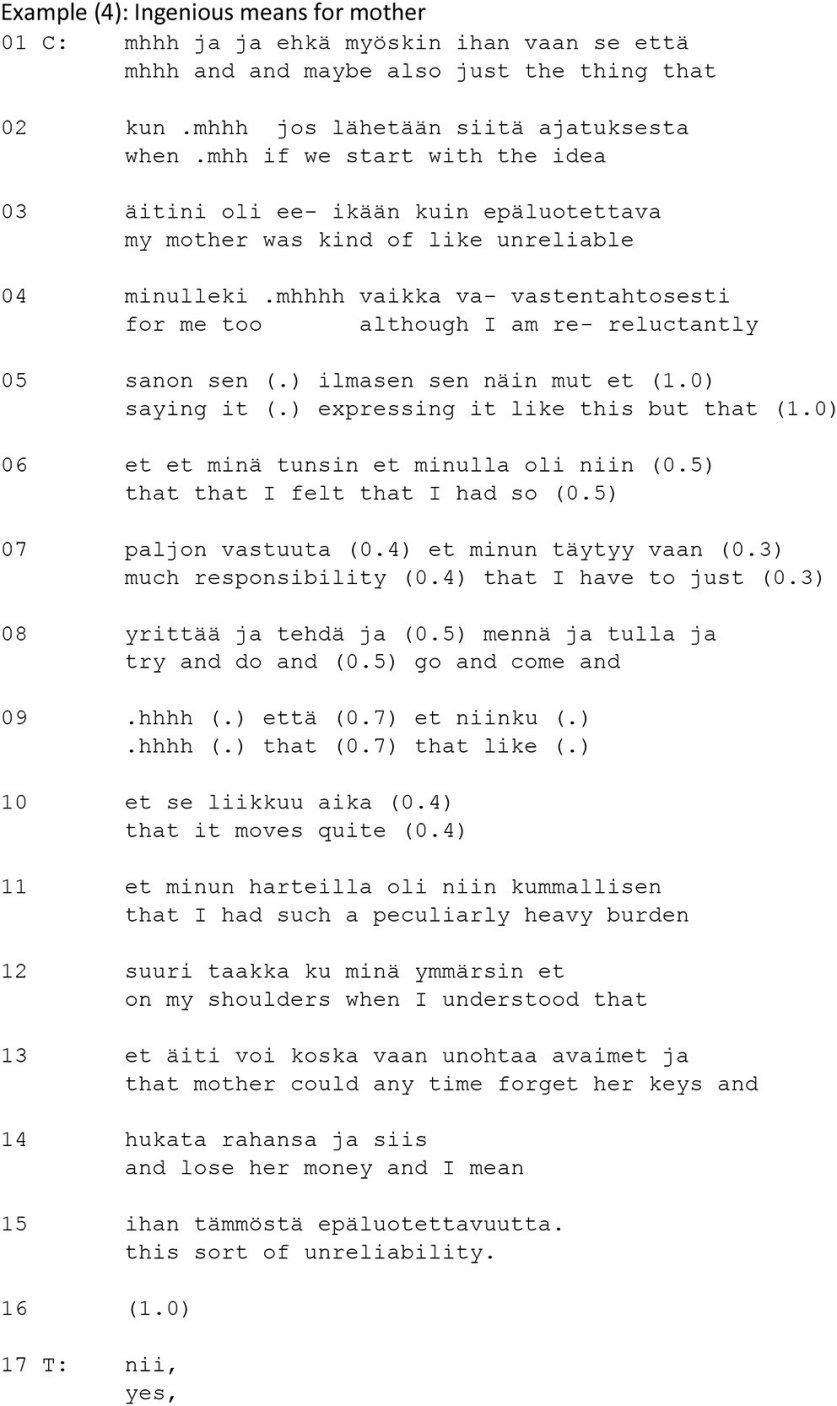


In her account, the client uses the first-person form when describing her experience (lines 5–7, 11, 19). Unlike the previous extract where the client used the zero-person form, here the client does not invite an empathetic recognition of her experience as strongly; rather, by reflecting on her childhood experience, she offers a place for an interpretation by the therapist. In his response (lines 23–25, 27), the therapist offers an interpretation of the client's experience that differs in content and perspective from that offered by the client herself. The therapist's turn begins with the particle *et* (“that”) (line 23), which marks that the turn is based on the contents of the client's previous turn. This is followed by a zero person construction with a modal verb *vois kääntää… ja ajatella* (“Ø [one] could turn… and think”) (lines 23–24). Thus, the design of the turn does not specify whether the person who could turn the thought around is the therapist or the client. The zero-person offers the client a slot where she can examine her thoughts from the therapist's perspective. In other words, while the therapist is the one who delivers the interpretation, through the zero-person he offers the place of the analyzer/examiner of the past situation to the client and so distributes the agency in interpreting the experience between himself and the client.

The interpretation changes the perspective and suggests that the motivation for the client's childhood behavior came from the client's mother instead of the client. The client initiates a partial confirmation with a concessive phrase, *niin voishan sen niinkin* (“yes Ø [one] could also think it so”), also using the zero form (line 29), and so takes the interpretive position offered by the therapist. The therapist intervenes in this (line 30) with a turn that is marked as a continuation of his previous turn by the particle *ja* (“and”). This turn presents the client with the consequences of her mother's behavior: it offers the experience [“no time to think about (one's)self nor to live Ø (one's) own life”] to the client to identify with. The client then produces an apparent confirmation (line 33). It begins with an agreeing particle, *nii* (“yes”), followed by an *et* (“so that”) initiating elaborating talk where the focus is on her mother. Nonetheless, the content of the talk is in slight contradiction to the therapist's suggestion in lines 23–25. However, the client maintains a reflective position on her experience and so aligns with the therapist's interpretative project that he suggested through the zero-person form.

The two examples above illustrated the two contexts in which the therapists in our data use the zero-person in their responses to the client's third-party complaints. In the empathetic response, the zero-person treated the client's experience as recognizable to the therapist. In the interpretative response, the therapist presented his own interpretation of the client's experience, and, by using the zero-form, invited the client to share the interpretative position. In contrast to these two uses of the zero-form, we will next discuss cases where the therapist refers to the client with the second person form.

### Creating Asymmetric Agency: Second Person Reference

Whereas, the zero-person in therapists' formulations invites patients to deal with their experience, either emotionally or by taking an analytic stance toward it, we found that second person references are used for re-constructing the client's past history. We argue that in these cases, the use of the second person singular pronoun treats the client's experience (in relation to third parties) as not shared by the client and the therapist, thus displaying asymmetric epistemic access to the experience (cf. the zero-form). Instead of providing an empathetic response to or an interpretation of the client's previous account, turns that include a second person singular pronoun make relevant a further explication of the experience.

Example (5) provides a case in point. The example is from cognitive therapy, where the client has discussed a recent meeting with her father (referred to as *Matti*) in which her father had described her mother's behavior as outrageous when she had been a child. At the beginning of the extract, the client moves to discuss the conflict of loyalty she feels between her divorced parents.


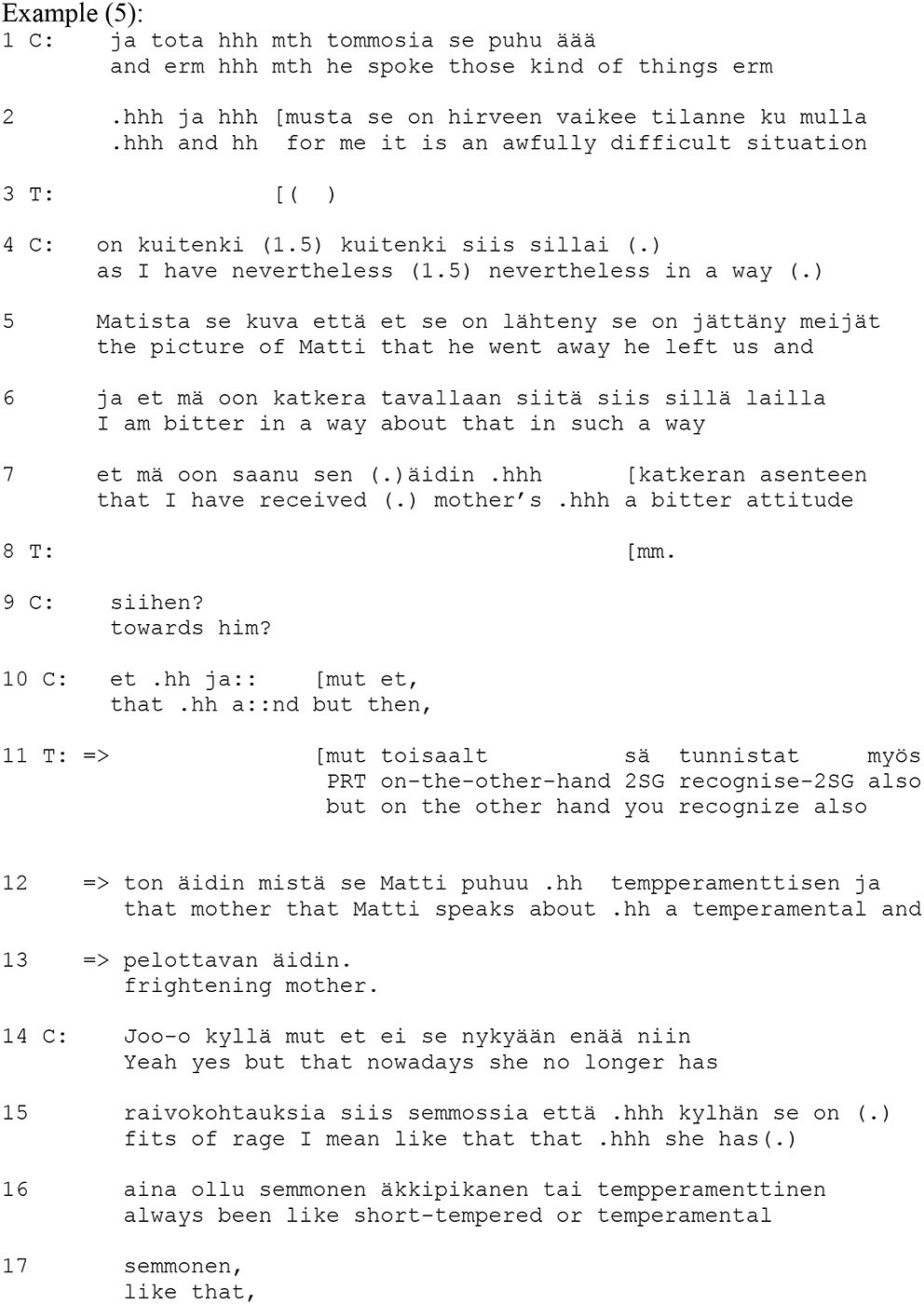


In her account, the client uses the first-person form “I” (lines 2, 4, 6–7). As the client does not use the zero-person form, she does not invite as strong an empathetic recognition of her experience as in Example 3. Instead, by reflecting on her childhood experience, the client offers a place for an interpretation by the therapist. The therapist responds to the client's account by highlighting the other side of her conflict of loyalty, that is, her mother and her unpredictable behavior (lines 11–13). The turn begins with a contrastive conjunction *mut* (“but”) followed by the adverb *toisaalta* (“on the other hand”), followed by a declaratively formulated B-event statement that concerns the client's perception *sä tunnistat myös* (“you also recognize”). B-event statements, namely declaratively formulated utterances that fall into the recipient's knowledge domain, function as polar questions by making a confirmation or disconfirmation the relevant next turn (Labov and Fanshel, 1977). The beginning of the therapist's turn (“but on the other hand”) maintains the relevance of the client's experience—as recounted by the client—but the rest of the turn suggests that there is another side to the client's story. The therapist's assertion is thus empathetic toward the client's description, but it also confronts the client's talk by bringing in elements that have not been mentioned explicitly, namely, the client's negative attitude toward her mother. By formulating her turn as a B-event statement with a second person singular pronoun, referring directly to the client as “you,” the therapist indicates more of an epistemic asymmetry between the participants than in the previous examples, where the zero-person form suggested a shared affective or interpretative stance. Furthermore, compared to the zero-person form, which implies shared agency, the use of “you” also evokes “I,” and thus two separate agents in the on-going situation.

In comparison to the uses of the zero-person in the context of empathy, here the client's experience is construed as belonging to the client's domain of knowledge, as something that the therapist can infer but to which she lacks equal epistemic access. This invites the client to relate herself to the therapist's suggestions and consider the ways in which she can, or cannot, agree with them. The client's response (line 14) begins with a conjunction chain *joo kyllä mut* (“yeah yes but”), whose basic function is to claim that the other speaker has incorrect or insufficient knowledge (Niemi, 2014). In this way, the client also implies epistemic asymmetry between the participants. This is followed by talk that returns to the present: the client's mother no longer suffers from outbursts of rage. The client uses the characterization “temperamental” (already used by the therapist in line 12), which is quite different from, and in a way more complimentary than, the characterization “getting sudden and frightening fits of rage.”

In sum, unlike the empathetic responses with the zero-person, here the experience in question (a negative stance toward the client's mother) is not displayed as being equally accessible to the participants; rather, the therapist seeks to help the client identify all the aspects of the past experience that need to be dealt with in the therapy session. As we claimed in the analysis of the previous extracts, shared agency and shared epistemic stance are bounded in the use of the zero-person form. For shared agency, the participants require shared knowledge. When using the second person form, the therapist implies that there is insufficient shared knowledge for shared agency to be attributed when describing the experience.

The next extract (6) further elaborates on the therapist's use of second person references. It comes from the same therapy session as Example (4) and it is a direct continuation of it.


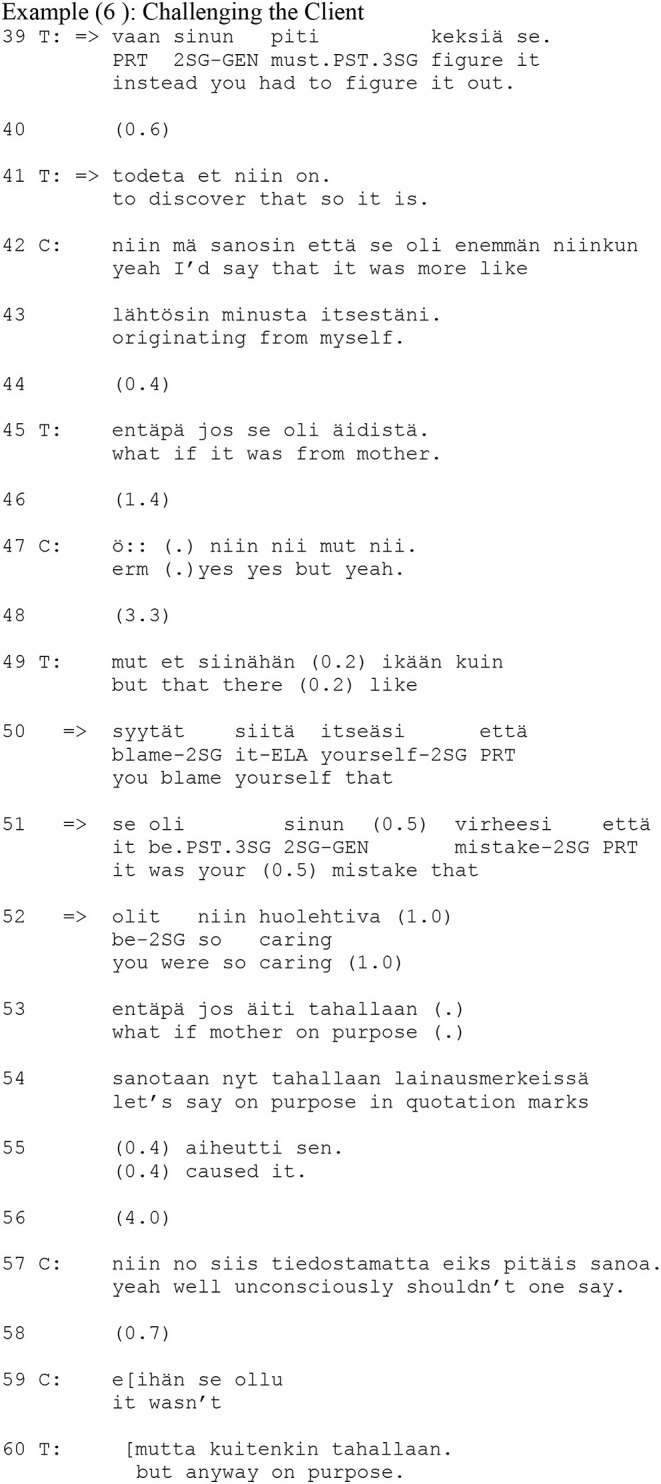


In response to the client's elaboration after the interpretation in Example (4), the therapist sums up the meaning of the client's mother not telling the client that she should take care of things: the client had to figure it out for herself (line 39). The turn begins with the conjunctive adverb *vaan* (“instead”), which makes the turn a direct syntactic continuation of the client's previous turn. Consequently, the turn aligns grammatically with the client's previous turn, but it shifts the focus from the mother to the client. Moreover, the turn includes the modal verb *piti* (“had to”), expressing necessity. The modal verb construes the past situation as a burden to the client and already implies that this necessity perhaps came from the client's mother (which the therapist in his later turn in line 45 explicitly suggests). Again, through the second person reference, an asymmetry between “you” and “I” is established; the therapist (first in lines 49–55 and then in line 60) infers aspects of the clients past experience that have not been made available in the previous talk but have to be traced. Here, the therapist does not invite the client to co-interpret the experience but instead suggests, and later in line 60 insists on, an interpretation, regardless of the client's resistance (first in lines 42–43 and later in lines 57–59).

The client's response begins with the particle *nii no* (“yeah well”), which implies disagreement. It is followed by talk that transforms the therapist's suggestion that the client's mother “on purpose,” (*tahallaan*, line 54) caused the client's over-developed sense of responsibility into a view that is more favorable toward the client's mother: she did it “unconsciously” (*tiedostamatta*). As we can see from the continuation (lines 59, 60), this is followed by a sequence where the client and the therapist disagree on the real state of affairs in the past. Unlike the extracts where the therapist used the zero-person form, the therapist does not suggest that the client's experience is available in the here-and-now of the therapeutic interaction; rather, there is a gap between the therapist's and the client's knowledge. However, in her response in line 57, the client uses the zero-person form and so suggests a more shared agency and a more symmetric epistemic relationship between the participants when interpreting the experience in psychotherapeutic terms.

To sum up, the second person reference was used in situations where the client's experience and emotion were not dealt with as mutually accessible and shared but as an experience that must be traced from the client's life history. The therapists' turn with the second person reference re-constructed the client's life history from the therapist's perspective, offering a version that differed from the client's account for the client to evaluate. By doing this, they challenged the client's previous understanding. The asymmetry that the person reference builds between “you” and “I” (in contrast to a shared understanding of emotion or interpretive agency in the uses of the zero-person) can be seen to reflect this epistemic difference and distance from the experience.

### Summary of the Results

In the four examples above (3–6), we discussed the use of personal forms in three types of therapist responses: a zero-person form displaying empathy, a zero-person form inviting interpretation, and a second person form in the context of tracing the problematic elements of the client's life history. We proposed that these forms perform distinct functions in therapeutic interaction. The use of the zero-person in general creates symmetry between the participants. When used in the context of empathy, it displays access to and understanding of the experience described by the client. Thus, it creates a symmetric relationship toward the emotive experience and functions as an empathetic response. Moreover, it invites the client to retake the “position of the zero”—the experiencing subject—and to notice and live through the emotive experience together with the therapist. When used in the context of interpretation, in turn, the zero person invites the client to adopt the position of an observing subject together with the therapist. In this use, it aims to create distance between the experienced emotion and the client and invites the client to take an interpretive perspective on the experience together with the therapist. The use of the second person form “you” always invokes the speaking “I” (Benveniste, 1966 [1956]) and thus foregrounds the separateness of the knowledge and agency of the participants. In our cases, the therapist uses the second person form in turns where s/he names an emotion or experience that has been implied but not previously named in the client's talk. The use of this form thus explains the client's experiences from the therapist's perspective and aims to identify the experience to be dealt with in therapy.

## Discussion

As Enfield (2013) observes, social agency is a dynamic, interactional phenomenon. Our analysis demonstrates that this perspective is also applicable to and informative for the study of psychotherapeutic process. The strength of the conversation analytic and interactional linguistic approaches adopted in this research lies in their efforts to study agency as a two-way relationship distributed over the course of actions performed by the therapist and client during therapeutic sessions. Thus, the analysis centers neither solely on the inner processes occurring within the client nor on the interventions performed by the therapist. Instead, the focus of analysis is the joint negotiation of agency and the consequent transformation of the description of the client's experience (see also Peräkylä, 2019).

Our analysis indicates that, when several options exist for person reference in a language, psychotherapists' choice of a particular alternative gains its specific meaning in its interactional context. In the case of third person complaints, the use of the zero-person form, which has previously been described as a “vague” person reference (Kurri and Wahlström, 2007), did not merely imply weak agency for the client (or the therapist); rather, it indexed the sharedness of the experience and agency in the contexts of empathy and interpretation. In comparison to the zero-person form, the choice of the second person singular pronoun placed the client more clearly ‘on stage’ as the target of the talk. This separateness, too, performs therapeutic functions. In the context of responses to third party complaints, the separateness of the agents (therapist and client) served to trace or identify the experience to be dealt with, in order to make the experience mutually available here and now. By contrast, in the situations where the therapist used the zero-person form, the person reference did not objectify the client as an agent under someone else's surveillance; instead, it implied the boundedness of the agents. In other words, the experience was not scrutinized from outside but within the slot opened up by the zero-person.

Our study also indicates that in Finnish psychotherapy sessions, the use of the zero-person distributes accountability and responsibility for the emotive experience between the therapist and the client. In that way, it invites the client to relate with the emotive experience in the here-and-now of the therapy session. Moreover, the use of the zero-person in interpretative actions to distribute accountability and responsibility for an action invites the client to construct an interpretative position toward the past experience in question. This, in turn, may help clients first to re-interpret the distribution of agency (accountability and responsibility) of their experiences and to adopt an interpretative perspective in the future: to analyze the distribution of accountability and responsibility in their lives and adopt an agentive position with respect to their decisions.

Our study specifies earlier research on agentless talk (Kurri and Wahlström, 2007). In contrast to that research, our study demonstrated that “vague” person references by therapists in the context of empathy and interpretation do not simply attempt to save the client's face; rather, they distribute agency between the client and the therapist. By using a zero-person form in these contexts, the therapists offered their clients a position to identify with, a position of empathetic understanding and interpretation of their experience (e.g., Eagle and Wolitzky, 1997; Greenberg and Elliott, 1997).

Our data comes from Finnish psychotherapy interactions which does not mean that the distribution of agency would be a uniquely Finnish phenomenon (see, e.g., Enfield and Kockelman, 2017, Rossi and Zinken, 2016). This raises interesting questions on how the distribution of agency is accomplished in other languages since person systems in languages organize person in different ways (Siewierska, 2004, Malchukov and Siewierska, 2011). The study of distribution of agency in therapeutic interaction opens thus an avenue to comparative studies on how cultural and language differences manifest in psychotherapeutic work.

In this study, the focus of analysis was the detailed ways in which personal forms are used in real time therapeutic interaction and therapists' use of these linguistic means for subtly distributing agency between themselves and the client. In this respect, we found no differences between the two therapeutic approaches (cognitive therapy and psychoanalysis). It should be born in mind, however, that our dataset contained just one therapist from each approach, and thus the question of differences between therapeutic approaches in the distribution of agency remains for further research.

## Data Availability Statement

The raw data supporting the conclusions of this article will be made available by the authors, without undue reservation.

## Ethics Statement

Ethical review and approval was not required for the study on human participants in accordance with the local legislation and institutional requirements. The patients/participants provided their written informed consent to participate in this study. Written informed consent was obtained from the individuals for the publication of any potentially identifiable images or data included in this article.

## Author Contributions

ME, LV, and EW: conception and design of the study, acquisition, analysis, interpretation of data, and drafting and revising the manuscript. All authors contributed to the article and approved the submitted version.

## Conflict of Interest

The authors declare that the research was conducted in the absence of any commercial or financial relationships that could be construed as a potential conflict of interest.

## Glossary

2: Second person
3: Third person
Ø: Zero person
ACC: Accusative case
CLI: Clitic
COND: Conditional
GEN: Genitive case
ELA: Elative case
ESS: Essive case
INF: Infinitive
NEG: Negation
PAR: Partitive case
PL: Plural
PRT: Particle
PST: Past
SG: Singular
